# Supplementary material for: Analysis of the alpha activity envelope in electroencephalography in relation to the ratio of excitatory to inhibitory neural activity
Source: PLoS One. 2024 Jun 13;19(6):e0305082. doi: 10.1371/journal.pone.0305082 (PMC11175473; doi:10.1371/journal.pone.0305082)
Supplement: S1 Table — (DOCX) [file pone.0305082.s001.docx]

**Supporting Information**

S1 Table. Information of all participants.

| Participant No. | Gender | Age | Group | MMSE | Alpha peak frequency | | Start | End |
| --- | --- | --- | --- | --- | --- | --- | --- | --- |
|  |  |  |  |  | O1 | O2 |  |  |
| sub-001 | F | 57 | AD | 16 | 9 | 8 | 140 | 200 |
| sub-002 | F | 78 | AD | 22 | 9 | 9 | 180 | 240 |
| sub-003 | M | 70 | AD | 14 | 9 | 9 | 40 | 100 |
| sub-004 | F | 67 | AD | 20 | 8 | 8 | 260 | 320 |
| sub-005 | M | 70 | AD | 22 | 9 | 9 | 400 | 460 |
| sub-006 | F | 61 | AD | 14 | 11 | 11 | 0 | 60 |
| sub-007 | F | 79 | AD | 20 | 9 | 8 | 160 | 220 |
| sub-008 | M | 62 | AD | 16 | 9 | 9 | 200 | 260 |
| sub-009 | F | 77 | AD | 23 | 11 | 10 | 120 | 180 |
| sub-010 | M | 69 | AD | 20 | 11 | 9 | 220 | 280 |
| sub-011 | M | 71 | AD | 22 | 10 | 10 | 280 | 340 |
| sub-012 | M | 63 | AD | 18 | 9 | 9 | 240 | 300 |
| sub-013 | F | 64 | AD | 20 | 11 | 11 | 40 | 100 |
| sub-014 | M | 77 | AD | 14 | 9 | 9 | 160 | 220 |
| sub-015 | M | 61 | AD | 18 | 9 | 9 | 0 | 60 |
| sub-016 | F | 68 | AD | 14 | 9 | 9 | 180 | 240 |
| sub-017 | F | 61 | AD | 6 | 11 | 10 | 80 | 140 |
| sub-018 | F | 73 | AD | 23 | 8 | 8 | 200 | 260 |
| sub-019 | F | 62 | AD | 14 | 10 | 10 | 100 | 160 |
| sub-020 | M | 71 | AD | 4 | 9 | 9 | 180 | 240 |
| sub-021 | M | 79 | AD | 22 | 9 | 9 | 200 | 260 |
| sub-022 | F | 68 | AD | 20 | 9 | 9 | 140 | 200 |
| sub-023 | M | 60 | AD | 16 | 9 | 9 | 180 | 240 |
| sub-024 | F | 69 | AD | 20 | 9 | 9 | 300 | 360 |
| sub-025 | F | 79 | AD | 20 | 9 | 9 | 180 | 240 |
| sub-026 | F | 61 | AD | 18 | 9 | 9 | 200 | 260 |
| sub-027 | F | 67 | AD | 16 | 9 | 9 | 180 | 240 |
| sub-028 | M | 49 | AD | 20 | 9 | 9 | 360 | 420 |
| sub-029 | F | 53 | AD | 16 | 9 | 9 | 0 | 60 |
| sub-030 | F | 56 | AD | 20 | 9 | 9 | 200 | 260 |
| sub-031 | F | 67 | AD | 22 | 10 | 11 | 100 | 160 |
| sub-032 | F | 59 | AD | 20 | 9 | 9 | 0 | 60 |
| sub-033 | F | 72 | AD | 20 | 10 | 10 | 80 | 140 |
| sub-034 | F | 75 | AD | 18 | 8 | 8 | 120 | 180 |
| sub-035 | F | 57 | AD | 22 | 9 | 9 | 20 | 80 |
| sub-036 | F | 58 | AD | 9 | 11 | 9 | 160 | 220 |
| sub-037 | M | 57 | NC | 30 | 9 | 9 | 220 | 280 |
| sub-038 | M | 62 | NC | 30 | 10 | 10 | 280 | 340 |
| Participant No. | Gender | Age | Group | MMSE | Alpha peak frequency | | Start | End |
|  |  |  |  |  | O1 | O2 |  |  |
| sub-039 | M | 70 | NC | 30 | 9 | 9 | 140 | 200 |
| sub-040 | M | 61 | NC | 30 | 9 | 9 | 400 | 460 |
| sub-041 | F | 77 | NC | 30 | 11 | 11 | 20 | 80 |
| sub-042 | M | 74 | NC | 30 | 10 | 10 | 220 | 280 |
| sub-043 | M | 72 | NC | 30 | 9 | 9 | 320 | 380 |
| sub-044 | F | 64 | NC | 30 | 11 | 11 | 100 | 160 |
| sub-045 | F | 70 | NC | 30 | 11 | 9 | 0 | 60 |
| sub-046 | M | 63 | NC | 30 | 9 | 9 | 0 | 60 |
| sub-047 | F | 70 | NC | 30 | 10 | 10 | 40 | 100 |
| sub-048 | M | 65 | NC | 30 | 9 | 9 | 60 | 120 |
| sub-049 | F | 62 | NC | 30 | 10 | 10 | 140 | 200 |
| sub-050 | M | 68 | NC | 30 | 8 | 9 | 45 | 105 |
| sub-051 | F | 75 | NC | 30 | 10 | 10 | 100 | 160 |
| sub-052 | F | 73 | NC | 30 | 10 | 10 | 120 | 180 |
| sub-053 | M | 70 | NC | 30 | 98 | 9 | 120 | 180 |
| sub-054 | M | 78 | NC | 30 | 9 | 10 | 60 | 120 |
| sub-055 | M | 67 | NC | 30 | 11 | 11 | 360 | 420 |
| sub-056 | F | 64 | NC | 30 | 9 | 9 | 260 | 320 |
| sub-057 | M | 64 | NC | 30 | 11 | 11 | 120 | 180 |
| sub-058 | M | 62 | NC | 30 | 9 | 9 | 240 | 300 |
| sub-059 | M | 77 | NC | 30 | 9 | 9 | 140 | 200 |
| sub-060 | F | 71 | NC | 30 | 9 | 8 | 200 | 260 |
| sub-061 | F | 63 | NC | 30 | 10 | 10 | 200 | 260 |
| sub-062 | M | 67 | NC | 30 | 9 | 9 | 0 | 60 |
| sub-063 | M | 66 | NC | 30 | 10 | 10 | 100 | 160 |
| sub-064 | M | 66 | NC | 30 | 10 | 10 | 360 | 420 |
| sub-065 | F | 71 | NC | 30 | 10 | 10 | 180 | 240 |
| sub-066 | M | 73 | FTD | 20 | 8 | 8 | 100 | 160 |
| sub-067 | M | 66 | FTD | 24 | 12 | 12 | 220 | 280 |
| sub-068 | M | 78 | FTD | 25 | 8 | 9 | 80 | 140 |
| sub-069 | M | 70 | FTD | 22 | 8 | 8 | 180 | 240 |
| sub-070 | F | 67 | FTD | 22 | 9 | 9 | 140 | 200 |
| sub-071 | M | 62 | FTD | 20 | 9 | 11 | 280 | 340 |
| sub-072 | M | 65 | FTD | 18 | 8 | 8 | 120 | 180 |
| sub-073 | F | 57 | FTD | 22 | 9 | 9 | 120 | 180 |
| sub-074 | F | 53 | FTD | 20 | 9 | 9 | 240 | 300 |
| sub-075 | F | 71 | FTD | 22 | 11 | 11 | 300 | 360 |
| sub-076 | M | 44 | FTD | 24 | 9 | 9 | 120 | 180 |
| sub-077 | M | 61 | FTD | 22 | 9 | 10 | 140 | 200 |
| sub-078 | M | 62 | FTD | 22 | 9 | 9 | 160 | 220 |
| sub-079 | F | 60 | FTD | 18 | 10 | 10 | 280 | 340 |
| sub-080 | F | 71 | FTD | 20 | 10 | 10 | 440 | 500 |
| sub-081 | F | 61 | FTD | 18 | 10 | 10 | 200 | 260 |
| sub-082 | M | 63 | FTD | 27 | 9 | 9 | 160 | 220 |
| Participant No. | Gender | Age | Group | MMSE | Alpha peak frequency | | Start | End |
|  |  |  |  |  | O1 | O2 |  |  |
| sub-083 | F | 68 | FTD | 20 | 9 | 9 | 340 | 400 |
| sub-084 | F | 71 | FTD | 24 | 8 | 8 | 260 | 320 |
| sub-085 | M | 64 | FTD | 26 | 9 | 9 | 120 | 180 |
| sub-086 | M | 49 | FTD | 26 | 10 | 10 | 60 | 120 |
| sub-087 | M | 73 | FTD | 24 | 10 | 11 | 140 | 200 |
| sub-088 | M | 55 | FTD | 24 | 10 | 10 | 100 | 160 |

NC: Normal control; AD: Alzheimer's disease; FTD, frontotemporal dementia

MMSE: Mini-Mental State Examination

Start and End: 60 seconds extracted from original data
