# Supplementary material for: Analysis of the alpha activity envelope in electroencephalography in relation to the ratio of excitatory to inhibitory neural activity
Source: PLoS One. 2024 Jun 13;19(6):e0305082. doi: 10.1371/journal.pone.0305082 (PMC11175473; doi:10.1371/journal.pone.0305082)
Supplement: Attachment — Submitted filename: 08_Responses_R2.docx [file pone.0305082.s003.docx]

Responses to the reviewers

Thank you very much for your very thoughtful peer review.

We have again revised the manuscript carefully according to the reviewer’s comments. In particular, we have added a diagram illustrating the concept of this study.

Reviewer #1: I first would like to commend the authors for the improvement in the manuscript. With that said, I believe there to be view minor points to address before being ready for acceptance. These are the following:

Abstract:
Line 34-39: I strongly recommend breaking the sentence up into two to increase readability. E.g., Second sentence starting line 37 "This was done in 36....."

The sentence has been revised.

The alpha wave envelope was subjected to secondary differentiation. This gave the positive (acceleration positive, Ap) and negative (acceleration negative, An) values of acceleration and their ratio (Ap-An ratio) at each sampling point of the envelope signals for 60 seconds. This analysis was performed on 36 participants with Alzheimer's disease (AD), 23 with frontotemporal dementia (FTD) and 29 age-matched healthy participants (NC) whose data were provided as open datasets.

Highlights:
Line 71: I recommend changing the starting of the second point so it varies slightly from the first (i.e., not starting with "a new" once again.

The highlight #2 has been changed.

#2: From the perspective of excitatory and inhibitory regulation, a novel aspect of cortical alpha oscillations was proposed

Introduction:
Line 91-98: Very well improved!

We would like to thank the reviewers for their help.

Line 105-108: Once again, a bit difficult to read. I recommend re-structuring/ splitting into two sentences.

The sentence has been revised and split into two sentences.

One hypothesis concerning the timing of the inhibitory and excitatory effects of alpha oscillations, including the waxing and waning phenomenon, proposed that the rhythmicity of alpha waves mediates inhibition [19-21,23]. Another suggested that waxing and waning resulted from the summation of multiple periodic phenomena [14].

Line 114: Move this to the sentence after, e.g., by stating "Such aims were based upon previous work, which defines.... (then state where it is based upon).

The sentences have been revised.

The aim of the present study was to show characteristics of alpha wave fluctuation at rest, i.e. 1) the ratio of increasing and decreasing forces that form the alpha oscillation and its relationship with amplitude, and 2) the difference of these values between healthy participants and patients with dementia in whom cognitive dysfunction occurred. These aims were based on previous work suggesting that the waxing and waning of alpha waves is regulated in an amplitude-dependent manner [24, 25] and that this functional regulation may be disrupted in patients with dementia [36-39].

Line 136: I would move this to the discussion/limitation section as keeping it in the introduction weakens the argument of the current exploration.

We have moved part of the sentence into the section of limitation.

(In Limitations)

We did not consider a direct correspondence with the NMMs or their equations in the present report, but a mathematical model to explain this phenomenon should also be considered.

Line 167: This sentence comes off weak. Perhaps saying "This study is believed...." rather than "thought". A minor recommendation but wording does strengthen the argument of the work to the reader!

Thank you for your suggestion. The sentence has been revised.

This study was believed that the present analysis would also allow us to detect the destruction of the function of alpha activity in various disorders.

Methods:
The authors have put in evident work to drastically improve the methods section, making it a lot more cohesive and sound. With that stated, I highly recommend adding some visual support (e.g., figures or diagrams) which support the research/analysis. As the concepts explore are fairly complex in nature, I strongly believe a visual component will strengthen receptivity of the work immensely.

We have added a figure which illustrated an overview the method of the present study.

I hope that these minor suggestions help improve the manuscript further beyond the already evident improvement!

Thank you again for your detailed peer review.

Reviewer #2: All comments were adequately addressed by the authors.
I would like to thank the authors for the patience taken to revise their work. I also suggest, as a future direction, to investigate the mechanisms underlying the acceleration/deceleration of the envelope in the alpha band, as it might provide an interesting window into neural activity.

Thank you for your kind comments. Thanks to your suggestions, we have been able to improve the manuscript. We will proceed to investigate the underlying mechanisms of this phenomenon and develop a mathematical model.
